# Supplementary material for: Suillin: A mixed-type acetylcholinesterase inhibitor from Suillus luteus which is used by Saraguros indigenous, southern Ecuador
Source: PLoS One. 2022 May 16;17(5):e0268292. doi: 10.1371/journal.pone.0268292 (PMC9109927; doi:10.1371/journal.pone.0268292)
Supplement: S1 File — (PDF) [file pone.0268292.s001.pdf]

Supplementary material

Suillin: a mixed-type acetylcholinesterase inhibitor from *Suillus luteus* which used by Saraguros indigenous, southern Ecuador

Andrade, José Miguel[1¶]; Pachar, Pamela[1]; Trujillo, Luisa[1], Cartuche Luis[1¶*]

[a] *Departamento de Química, Universidad Técnica Particular de Loja (UTPL), Loja, Loja, Ecuador.*

Corresponding author. Luis Cartuche, lecartuche@utpl.edu.ec,

Contents

1	**Table 1.** Rate of reaction of acetylcholinesterase against three concentrations of suillin
2	**Figure 1.** Inhibition model from suillin calculated from non-linear regresion analysis
3	**Table 2.** Non-linear regression analysis on suillin
4	**Table 3.** Rate of reaction of acetylcholinesterase against three concentrations of suillin at differnte molar concentration of substrate
5	**Figure 2**. Progresion curves for different concentrations of suillin with six concentrations of substrate
6	**Table 4.** Best enzymatic inhibition model for suillin calculated from non-linear regression analysis

Table 1. Rate of reaction of acetylcholinesterase against three concentrations of suillin

µM Suillin	Vo mM.min^{-1}		
1000	0.35383	0.2543	0.3132
100	4.4906	5.223	5.0353
10	28.696	30.39	29.543
1	33.798	34.376	34.438

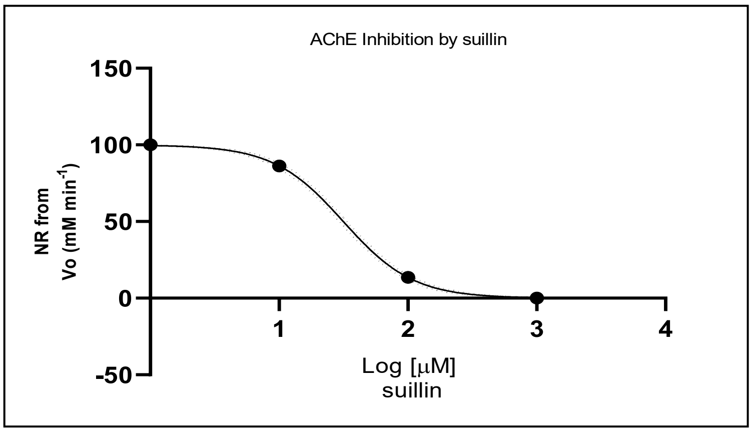

Figure 1. Inhibition model from suillin calculated from non linear regresion analysis
NR from Vo. Normalized ratio from initial velocity in mM/min

Table 2. Non linear regression analysis on suillin
log(inhibitor) vs. normalized response -- Variable slope

Best-fit values

LogIC50	1.498
HillSlope	-1.603
IC50	31.5
Std. Error	
LogIC50	0.01264
HillSlope	0.04037

95% CI (profile likelihood)

LogIC50	1.470 to 1.526
HillSlope	-1.697 to -1.517
IC50	29.51 to 33.61

Goodness of Fit

Degrees of Freedom	10
R squared	0.9992
Sum of Squar[illegible]	18.15
Sy.x	1.347

Number of points

# of X values	12
# Y values analyzed	12

Table 3. Rate of reaction of acetylcholinesterase against three concentrations of suillin at different molar concentration of substrate

mM [S] ATChI	0 μM			30 μM suillin		
0.25	10.257	11.047	9.9617	4.2157	4.3971	5.4102
0.5	15.715	15.608	16.222	8.3033	8.5943	8.1515
1	21.426	24.634	24.778	9.2797	9.9243	10.569
1.5	24.4515	28.818	26.085	15.325	14.508	12.244
2	30.727	29.842	27.631	16.633	15.98	14.98
2.5	29.879	28.908	29.714	15.145	16.145	17.145

ATChI. Acetylthiocholine iodide

Table 3. Continuation

mM [S] ATChI	40 μM suillin			50 μM suillin		
0.25	3.3414	4	3.3359	1.3377	3.0434	2.4492
0.5	5.6032	7.4315	6.5173	3.7623	4.3715	3.5633
1	7.2301	7.9884	7.6046	4.0163	4.1922	5
1.5	8.3518	8.38	9.0731	4.1326	5.114	4.155
2	10.14	10.546	10.611	4.8424	5.3449	4.8063
2.5	11.062	11.861	11.4615	7.3117	7.8343	7.573

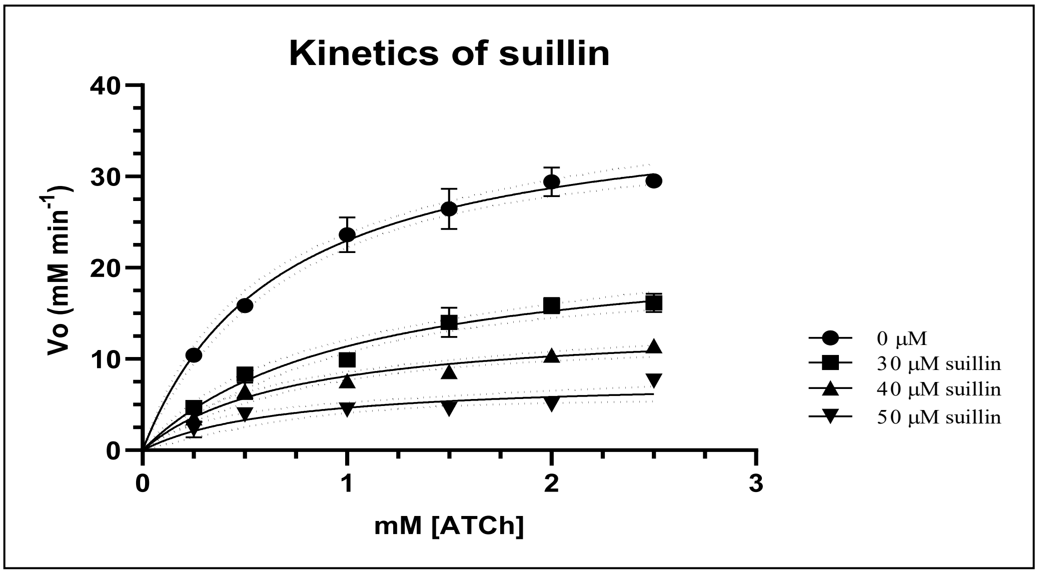

Figure 2. Progresion curves for different concentrations of suillin with six concentrations of substrate

Table 4. Best enzymatic inhibition model for suillin calculated from non-linear regression analysis

Graphpad Prism 8.0.1					Global (shared)
**Mixed model inhibition**					
**Best-fit values**					
Vmax	38.7	38.7	38.7	38.7	38.7
I	= 0.000	= 30.00	= 40.00	= 50.00	
Alpha	1.454	1.454	1.454	1.454	1.454
Ki	17.25	17.25	17.25	17.25	17.25
Km	0.6746	0.6746	0.6746	0.6746	0.6746
**Std. Error**					
Vmax	2.068	2.068	2.068	2.068	2.068
Alpha	0.6642	0.6642	0.6642	0.6642	0.6642
Ki	4.974	4.974	4.974	4.974	4.974
Km	0.1052	0.1052	0.1052	0.1052	0.1052
**Goodness of Fit**					
Degrees of Freedom					68
R squared	0.969	0.6776	0.9226	-1.599	0.9426
Sum of Squar	29	105.8	9.595	125.2	269.6
Sy.x					1.991
**Constraints**					
Vmax	Vmax > 0 and shared	Vmax > 0 and shared	Vmax > 0 and shared	Vmax > 0 and shared	
I	I = 0	I = 30	I = 40	I = 50	
Alpha	Alpha > 0 and shared	Alpha > 0 and shared	Alpha > 0 and shared	Alpha > 0 and shared	
Ki	Ki > 0 and shared	Ki > 0 and shared	Ki > 0 and shared	Ki > 0 and shared	
Km	Km > 0 and shared	Km > 0 and shared	Km > 0 and shared	Km > 0 and shared	
**Number of points**					
# of X values	18	18	18	18	
# Y values ana	18	18	18	18	
