# Supplementary material for: Suillin: A mixed-type acetylcholinesterase inhibitor from Suillus luteus which is used by Saraguros indigenous, southern Ecuador
Source: PLoS One. 2022 May 16;17(5):e0268292. doi: 10.1371/journal.pone.0268292 (PMC9109927; doi:10.1371/journal.pone.0268292)
Supplement: Attachment — Submitted filename: Response to reviewers.docx [file pone.0268292.s002.docx]

Feb, 2022

Emily Chenette

Editor in Chief

Public Library of Science PLOS One

**Subject:** Online Manuscript Submission

To Editor

I am submitting a required revision for consideration in PlosOne with the changes made in the manuscript, highlighted with the track changes mode, for all the suggestions made by reviewers as described below:

Reviewer 1:

This manuscript describes the isolation, structural analysis and action on acetylcholinesterase of suillin, a terpenoid (tetraprenylphenol) from the basidiomycete Suillus luteus. Suillin is a reversible non-competitive inhibitor of acetylcholinesterase. This compound is interesting from a mechanistic point of view, but likely not for a possible interest as anti-AD drug. However, the study is weak and the enzymologic part needs to be carefully reworked.

Answer:

We started changing the title of the manuscript to state the correct mode of action of suillin against acetylcholinesterase enzyme, after a carefully revision of the data. Analysis in the software Graph pad was remade to improve and support our conclusions. A comprehensive analysis of this results is included in section **3.3**. **Suillin kinetics** with updated and relevant references to support our conclusions

Major points

Title has to be changed: suillin is not a strong cholinesterase inhibitor (cf line 231, Table I, Ki = 22 microM)

Suillin is a mixed type inhibitor according to our results, with a moderate effect so, our title enclosure mainly the mode of action before the potency and is stated as follow:

**‘Suillin: a mixed type acetylcholinesterase inhibitor from *Suillus luteus* which used by Saraguros indigenous, southern Ecuador’**

Introduction: Line 70, AChE is not only mainly found at neuromuscular junction but also in brain where it plays a key role.

Line 71-72 how high level of AChE at neuromuscular junctions can cause neurological disorders ?

Information was changed for:

Line 97…AChE, a cholinergic enzyme, is primarily found at postsynaptic neuromuscular junctions, especially in muscles and nerves, where it can terminate synaptic transmission. AChE inhibition results in accumulation of acetylcholine leading to increased stimulation of muscarinic and nicotinic receptors which provides symptomatic relief to memory deficit in Alazheimer’s disease (AD)

The main idea is to describe the mechanism of action of AChE and its neurotrasnmisor (Acetylcholine) and the role involved in AD.

Formula of suillin, Line 140: suillin is an acetylester, like aspirin and acetylcholine. Why the authors did not check whether this compound could be also substrate of acetylcholinesterase ? There are many examples of compounds acting both as a substrate and inhibitor of cholinesterases (ex, aprophen).

Our methodology relies on the releasing of a product and the interaction with a revealing reagent (Ellman’s reagent) that gives a colored product that can be measured spectrophotometrically. Further studies can be conducted to find this but, right now, it is impossible for us to measure this effect. A review of aprophen and the method employed to measure the inhibitory activity was included in the manuscript, to contrast our method with another more suitable to measure this kind of activity. Unfortunately, we do not have the same facilities to do it right now.

Lines 163 and 235 and Fig 4: AChE kinetics at concentrations varying between 2.5 and 25 mM cannot be described by the Michaelis-Menten model. Fig 4 shows the rate of AChE as a function of ATC ranging from 0.25 to 2.5 mM. The enzyme should be inhibited by excess substrate beyond 1 mM. How to explain that the enzyme is not inhibited by excess substrate ?

Several studies have been conducted with the methodology followed in this study, where M-M model fits perfectly with the ATC doses employed. Another consideration in our method is that the amount of enzyme emplyed is 50 mM to avoid the occurrence of false positives so, in this case, the amount needed of ATC to inhibit its activity is higher. According to Reiner and Simon (1977), the K_ss_ (substrate inhibition constat) for acetylthicoline is 14 mM. For that reason, our model is supported for several researches published with this conditions and by the cited reference above mentioned. (*Reiner E. Simeon V. 1977. Competition between substrates for acetylcholinesterase and cholinesterase. Biochem parmacol. 1985. 34 (12): 2063-2068*). This information was not included in methodology because is well documented.

Line 170: this model equation suppose that the inhibitor only affects Vmax. How to rule out that inhibitor does not affect Km or both Km and Vmax?

The data supported a mixed inhibition model for suillin so, information about the equation and some parameters were registered in section 3.3. Suillin kinetics.

Lines 221, 23: IC50 and Ki do not match as they should for pure non competitive inhibitor (as it is shown in Fig 4 insert, alpha =0): Ki = IC50.

The data supported a mixed inhibition model for suillin so, information about the equation and some parameters were registered in section 3.3. Suillin kinetics.

Minor points

Line 146 : Was MeOH the solvent of suillin for inhibition measurements ? (What was the final MeOH concentration in assay ?)

As a protic non-selective solvent, MeOH was selected to dissolve samples and employed as a negative control at a maximum concentration of 10% in the final mix volume without affecting enzyme reaction

Reviewer 2

1. Table 1 & Figure 2: Positive control for Ellman assay is missing. A known drug in inhibiting AChE activity should be included.

Donepezyl hydrochloride was used as a positive control, including a calculated IC_50_ in Table 1 and in the description of the methodology along with a previous report from our research group.

2. As several compounds were identified in the extracts. Why suillin was the major component in suppressing AChE? For other compounds identified, same experiments (i.e. Ellman assay and kinetic analysis) should also be conducted in order to compare and confirm suillin as the main functioning chemical.

All the compounds were tested in the assay at a concentration of 250 µM. Only suillin exhibited a remarkable activity below 250 µM and was included in the kinetic assay.

3. Many fungi have been found to have anti-cholinesterase function in previous studies, so the perspective of this study is not new. Also, if the authors would like to propose it as a candidate for neurodegenerative treatment, the scale of study in this manuscript is not adequate. More studies (e.g. in vitro) should be conducted to make the findings more reliable and persuasive.

Fungi in Ecuador have been poorly exploited and assessed for the identification of chemical compounds or relevant biological acitivities, for that reason, our research group is carrying out basic research to identify potential fungal candidates for applied research. *Suillus luteus* is a well-documented and recognized fungi, however, the data provided in this research contributes to the scientific knowledge to validate the pharmacological potential of the fungal diversity. A complete discussion and recommendation for further studies was included in the manuscript. The mechanistic approach conducted in this study for suillin is the beggining of a research field that we pursue.

Minor comments

1. Some typos need to be rectified. (e.g. ‘acethylcholinesterase’)

The whole manuscript has been carefully reviewd for spelling errors or language mistakes.

Final consideration.

Acknowledgment section was changed because there is no specific funding for this Project.

The authors support this reviewed version of the manuscript, and would like it to be appreciated in this important scientific journal. It is our first interaction with this journal and we would be very grateful if A. M. Abd El-Aty ([orcid.org/0000-0001-6596-7907](https://orcid.org/0000-0001-6596-7907), Cairo University, Egipt) participate as Academic editor four our work.

Yours sincerely,

*Prof Dr Luis Emilio Cartuche Flores*

*Departamento de Química*

*Universidad Técnica Particular de Loja,*

*P.O. Box 11 01 608, Loja - Ecuador,*

*Tel: +593 7 370 1444,*

*E-mail address: lecartuche@utpl.edu.ec*
