# Supplementary material for: Suillin: A mixed-type acetylcholinesterase inhibitor from Suillus luteus which is used by Saraguros indigenous, southern Ecuador
Source: PLoS One. 2022 May 16;17(5):e0268292. doi: 10.1371/journal.pone.0268292 (PMC9109927; doi:10.1371/journal.pone.0268292)
Supplement: Attachment — Submitted filename: Response to reviewers.docx [file pone.0268292.s003.docx]

April, 2022

Emily Chenette

Editor in Chief

Public Library of Science PLOS One

**Subject:** Online Manuscript Submission

To Editor

I am submitting a required revision for consideration in PlosOne with the changes made in the manuscript, highlighted with the track changes mode, for all the suggestions made by reviewers as described below:

Reviewer 1:

The manuscript was greatly improved. However, line 216 page 10, the formula used for determination of Ki is wrong. Is it a typo or a real mistake? Anyway, with such a "mistake" there is still doubt about the values of alpha and Ki.

Answer:

Formulas used in Graphpad packages for mixed model inhibition were included in the manuscript. There was a mistake not reviewing the formula when we submitted the last version of the document.

Reviewer #2: In this manuscript, the authors described their findings on suillin, the active ingredient extracted from the herb Suillus luteus can function as a mixed-type acetylcholinesterase inhibitor. The methods adopted in this study included thin-layer chromatography (TLC) to determine compounds from extracts of Suillus luteus, Ellman assay to characterize the extracts’ and suillin’s roles in inhibiting AChE and to conduct the kinetic analysis of suillin. As a conclusion, they proposed that suillin can be a candidate for treating neurodegenerative diseases. Although the most of problems have been addressed, there are still some questions:

- Table 1: IC50 of other chemicals isolated (i.e. linoleic acid, ergosterol etc.) should be included for comparison. What is the extraction efficiency of suillin from the herbal extract?

Answer:

Minor components were assessed at a maximum final concentration of 200 µM and no inhibitory effect was observed. To obtain IC_50_ values of such compounds we would need sample solutions with concentrations higher than 2000 µM and for pharmaceutical purposes there is no relevance to assess them. However, we included some additional information in the manuscript for Linoleic acid as a neuroprotective agent to not dismiss the presence of it as an important isolated compound.

Line 255 states … Linoleic acid (LA), ergosterol and peroxyergosterol did not exhibited inhibitory activity at the maximum dose tested (200µM), however, dietary consumption of LA and other related PUFAs could be associated to a cholinergic transmission improvement, acting as neuroprotective agents in aged brains, where it has been demonstrated that the decrease of fatty unsaturated acids is associated to a decline in the neurological function [46].

- In the discussion, how mixed inhibitors to AChE can be discussed. Propose the mechanism of how suillin works on AChE, e.g. docking analysis.

A hypothesis of how suillin could be acting was included at the end of the manuscript with proper references. Docking analysis (Line 339) had been previously suggested for us to validate the mechanism of action proposed in our study but is matter of another possible future research. In vivo studies are also suggested.

Line 352 states.

Another possible explanation to the observed effect for suillin could be related to the peripheric anionic site (PAS) of acetylcholinesterase, a second substrate binding site, composed of five residues (Tyr70, Asp72, Tyr121, Trp279, and Tyr334) that lies at the entrance to the active site gorge and enhance the catalysis of Acetylcholine by trapping it to its way to the active site [51]. Suillin could be interacting with this site generating an ESI complex. Several compounds have been demonstrated to interact via PAS site, such as, edrophonium, which exerts a competitive reversible inhibition at a concentration of 0.1 µM and a mixed competitive-nonconmpetitive inhibitor at higher concentrations (1 to 10 µM) [52].

The authors support this reviewed version of the manuscript, and would like it to be appreciated in this important scientific journal. It is our first interaction with this journal and we would be very grateful if A. M. Abd El-Aty ([orcid.org/0000-0001-6596-7907](https://orcid.org/0000-0001-6596-7907), Cairo University, Egipt) participate as Academic editor four our work.

Yours sincerely,

*Prof Dr Luis Emilio Cartuche Flores*

*Departamento de Química*

*Universidad Técnica Particular de Loja,*

*P.O. Box 11 01 608, Loja - Ecuador,*

*Tel: +593 7 370 1444,*

*E-mail address: lecartuche@utpl.edu.ec*
